# Stereotactic radiosurgery for the treatment of melanoma and renal cell carcinoma brain metastases

**DOI:** 10.3892/or.2012.2139

**Published:** 2012-11-14

**Authors:** SHELLY LWU, PABLO GOETZ, ERIC MONSALVES, MANDANA ARYAEE, JULIUS EBINU, NORM LAPERRIERE, CYNTHIA MENARD, CAROLINE CHUNG, BARBARA-ANN MILLAR, ABHAYA V. KULKARNI, MARK BERNSTEIN, GELAREH ZADEH

**Affiliations:** 1 Division of Neurosurgery, University Health Network, University of Toronto, Toronto, Ontario, Canada; 2 Department of Radiation Oncology, University Health Network, University of Toronto, Toronto, Ontario, Canada; 3 Division of Neurosurgery, The Hospital for Sick Children, University of Toronto, Toronto, Ontario, Canada

**Keywords:** melanoma, renal cell carcinoma, brain metastases, stereotactic radiosurgery, local control

## Abstract

Renal cell carcinoma (RCC) and melanoma brain metastases have traditionally been considered radioresistant lesions when treated with conventional radiotherapeutic modalities. Radiosurgery provides high-dose radiation to a defined target volume with steep fall off in dose at lesion margins. Recent evidence suggests that stereotactic radiosurgery (SRS) is effective in improving local control and overall survival for a number of tumor subtypes including RCC and melanoma brain metastases. The purpose of this study was to compare the response rate to SRS between RCC and melanoma patients and to identify predictors of response to SRS for these 2 specific subtypes of brain metastases. We retrospectively reviewed a prospectively maintained database of all brain metastases treated with Gamma Knife SRS at the University Health Network (Toronto, Ontario) between October 2007 and June 2010, studying RCC and melanoma patients. Demographics, treatment history and dosimetry data were collected; and MRIs were reviewed for treatment response. Log rank, Cox proportional hazard ratio and Kaplan-Meier survival analysis using SPSS were performed. A total of 103 brain metastases patients (41 RCC; 62 melanoma) were included in the study. The median age, Karnofsky performance status score and Eastern Cooperative Oncology Group performance score was 52 years (range 27–81), 90 (range 70–100) and 1 (range 0–2), respectively. Thirty-four lesions received adjuvant chemotherapy and 56 received pre-SRS whole brain radiation therapy. The median follow-up, prescription dose, Radiation Therapy Oncology Group conformity index, target volume and number of shots was 6 months (range 1–41 months), 21 Gy (range 15–25 Gy), 1.93 (range 1.04–9.76), 0.4 cm^3^ (range 0.005–13.36 cm^3^) and 2 (range 1–22), respectively. Smaller tumor volume (P=0.007) and RCC pathology (P=0.04) were found to be positive predictors of response. Actuarial local control rate for RCC and melanoma combined was 89% at 6 months, 84% at 12 months, 76% at 18 months and 61% at 24 months. Local control at 12 months was 91 and 75% for RCC and melanoma, respectively. SRS is a valuable treatment option for local control of RCC and melanoma brain metastases. Smaller tumor volume and RCC pathology, predictors of response, suggest distinct differences in tumor biology and the extent of radioresponse between RCC and melanoma.

## Introduction

Renal cell carcinoma (RCC) and melanoma brain metastases have traditionally been considered ‘radioresistant’ to conventional fractionated external beam radiotherapy and external beam whole brain radiation therapy (WBRT) (1–4). In the past decade, stereotactic radiosurgery (SRS) has become a well-established treatment modality for local control for a number of tumor subtypes (5,6). The typically spherical, well-circumscribed morphology of brain metastases provide ideal targets for SRS. One of the theoretical benefits of SRS is its potential to overcome ‘radioresistance’ by delivering a single fraction of high dose radiation to the hypoxic tumor core with a sharp dose fall-off in the adjacent cells (7). Given the traditional understanding that melanoma and RCC patients are resistant to radiation therapy, recent literature have focused on the role of SRS for local control of RCC and melanoma brain metastases; the limited data support a favorable response to SRS with better local control and improved survival (8,9) We therefore aimed to evaluate our institutional results using SRS for treating RCC and melanoma brain metastases, with a focus on identifying predictors of response to achieve local control. We also compared the 2 tumor subtypes to determine whether there is a differential response to SRS.

## Materials and methods

### Ethics

This study was approved by the University Health Network Research Ethics Board of the University of Toronto.

### Patients and setting

We retrospectively reviewed a prospectively maintained database of all patients with brain metastases treated at the University of Toronto Gamma Knife (Elekta Instruments, Atlanta, GA) facility, from October 2007 to June 2010. All patients were assessed and monitored at the UHN Multidisciplinary Brain Metastasis Clinic staffed by neurosurgeons, radiation and medical oncologists. Patients were eligible to participate in the study if they had documented treatment data and clinical and radiological follow-up.

### Radiosurgery treatment protocol

On the day of treatment, the Leksell frame (Elekta AB) was applied to the patient’s head under local anesthesia. A high resolution gadolinium-enhanced MRI scan obtained the day prior to treatment was fused to the CT scan performed after frame placement. Using the GammaPlan (Elekta AB) software, the neurosurgeon, radiation oncologist and 2 medical physicists designed the dose plan. Doses were selected based on tumor size and location. Clinical and radiological follow-up after treatment typically occurred at 3-month intervals. Shorter follow-up intervals were performed if determined necessary by the treating physician.

### Data collection

Patient demographics, treatment history and clinical follow-up information were obtained from the UHN electronic medical records. All radiological imaging was reviewed by the first author (S.L.). Treatment response was categorized as stable (no change in size or smaller) or progression. Where there were uncertainties, the MRI report was reviewed. The time to progression for intracranial disease was defined as the time between SRS and the first follow-up MRI demonstrating lesion progression. Dosimetry parameters were collected using the GammaPlan software.

### Statistical analysis

Survival analysis was performed for time to progression of individual metastatic lesions. Progression-free survival estimates were determined using the Kaplan-Meier test. Exploratory univariate Cox proportional hazards regression analyses were used to identify independent variables associated with the local control. The proportional hazards assumption was confirmed by the inspection of partial residual plots as a function of time. To account for the fact that a number of patients harbored multiple lesions, the analyses were stratified based on the presence of multiple metastatic lesions. Given the limitations of the sample size, multivariate regression analysis was not performed. All analyses were performed using SPSS version 17 (SPSS, Inc., Chicago, IL).

## Results

### Patient population and dosimetry parameters

Of all patients treated with brain metastases at our institution between October 2007 and June 2010, 58 patients (25 RCC; 33 melanoma) were treated with Gamma Knife SRS. Thirty-six patients (7 females, 29 males; 16 RCC, 20 melanoma) with a total of 103 brain metastases (41 RCC, 62 melanoma) were included in the study (Table I). The median age was 52 years (range 27–81). The median Karnofsky performance status (KPS) score was 90 (range 70–100) and the median Eastern Cooperative Oncology Group (ECOG) performance score was 1 (range 0–2). Thirty-four of the lesions received adjuvant chemotherapy and 56 received pre-SRS WBRT. The median follow-up was 6 months (range 1–41 months).

The median prescription dose was 21 Gy (range 15–25 Gy) (Table II). The median Radiation Therapy Oncology Group (RTOG) conformity index was 1.93 (range 1.04–9.76). The median target volume was 0.4 cm^3^ (range 0.005–13.36 cm^3^). The median target minimum dose was 20.16 Gy (range 11.62–31.85 Gy). The median number of shots were 2 (range 1–22).

### Local control

**Figure 1:**
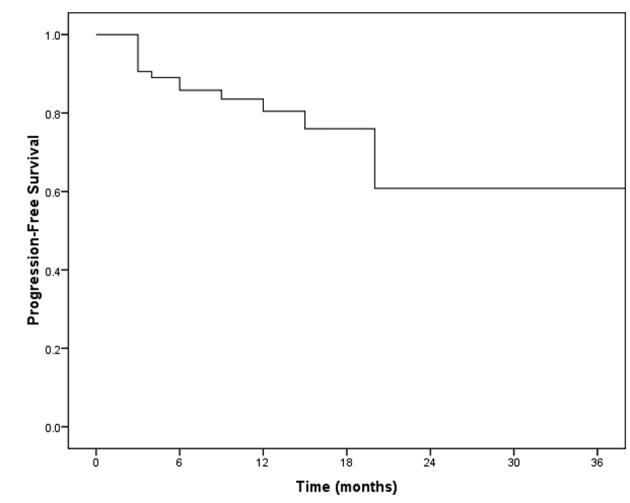
Kaplan-Meier survival curve

Actuarial local control for RCC and melanoma combined was 89% at 6 months, 84% at 12 months, 76% at 18 months and 61% at 24 months (Fig. 1, Table III). Local control at 12 months was 91 and 75% for RCC and melanoma, respectively.

Only 3 patients underwent surgical resection for treatment of local failure. Patients did not receive repeat SRS for treatment of local failure.

### Predictors of response

Smaller tumor volume (P=0.007) and RCC pathology (P=0.04) were found to be positive predictors of response in univariate Cox regression analysis (Table IV). Age, ECOG score, KPS score, adjuvant chemotherapy, pre-SRS WBRT and all SRS dosimetry parameters were not found to be significant variables.

Mean tumor volume of lesions that progressed was twice the size of lesions that remained stable after SRS treatment (2.62 vs. 1.27 cm^3^). Mean tumor volume of RCC metastases was smaller compared to that of melanoma metastases (1.28 vs. 1.54 cm^3^).

## Discussion

Local tumor control using SRS is a mainstay of the management for brain metastases. However, certain subtypes of brain metastases, specifically melanoma and RCC, have been considered resistant to SRS. Recent accumulating evidence supports the value of SRS for local control of melanoma and RCC, with a summary of published results presented in Table V. Our institutional experience with Gamma Knife SRS treatment of RCC and melanoma brain metastases also demonstrates that SRS is a valuable treatment option for local control for these tumor subtypes. Local control was better for RCC than for melanoma, suggesting a differential response to SRS between the 2 pathologies and a distinct difference in tumor biology. Positive predictors of response were smaller tumor volume and RCC primary pathology. Age, performance status, adjuvant chemotherapy and pre-SRS WBRT were not found to be significant variables. This highlights an important clinical decision-making point, that RCC and melanoma respond differentially to SRS.

We report 12-month local control rates of 91 and 75% for RCC and melanoma, respectively. These figures are comparable to what has been reported in the literature. Local control rates for melanoma range from 47 to 100% (8,10–19), while those for RCC range from 63 to 100% (8,9,15,20–26) (Table V). One of the challenges involved in gaining a better understanding from existing literature on this topic is that there is significant variability in how local control is defined and reported by individual groups in each study. A number of groups chose to report the actuarial local control rates at 6 or 12 months while others reported the local control rate based on the last radiological follow-up. Careful interpretation of existing literature is required and more reports of this nature adding to the body of literature with consistent criteria in assessing outcomes are necessary to gain a better understanding of specific tumor subtypes. Taking this factor into consideration, our results combined with reported data demonstrated that RCC and melanoma have a comparable local control rate when compared to other tumor subtypes, although RCC tends to demonstrate a better local control rate compared to melanoma.

The key finding of this study is that we determined that smaller tumor volume is a positive predictor of response for both RCC and melanoma to SRS. This is also supported by existing literature (14,17,18,26–28). Furthermore, we discovered that the mean tumor volume of lesions that had progressed was more than double that of lesions that remained controlled. Chang *et al*(10) reported that one year control rates for metastatic lesions less than and greater than 1 cm diameter (0.5 cm^3^) were 86 and 56%, respectively, using a minimum peripheral dose of 20 Gy for the majority of the lesions. This suggests that an aggressive approach for treating RCC and melanoma should be undertaken, with early intervention when the lesions are smaller. It also supports more frequent serial surveillance brain imaging for patients with RCC and melanoma to ensure early detection of tumor metastases at a smaller volume size. The radiobiology postulate in support of this would be a smaller total number of tumor cells with a smaller fraction of hypoxic tumor core which would therefore respond more effectively to SRS.

We identified RCC pathology to be a positive predictor of response to SRS. Lo *et al*(29) also discovered RCC pathology to be a positive predictor to SRS in comparison to melanoma metastasis. In our study, the mean volume of RCC metastases was smaller than that of melanoma metastases. Therefore it is possible that tumor volume was a contributing factor to RCC pathology being a positive predictor of response to SRS, and that tumor volume and tumor pathology may not be independent of each other. These data also reflect the propensity of RCC metastases to be smaller at the time of presentation to the treatment.

The small size and multiplicity of the metastases treated may have been reflective of the trend at the time at our center, which was to administer aggressive treatment of intracranial disease for radioresistant tumors in the setting of stable extracranial disease. This trend may have been in the context of obtaining control of intracranial diseases in order for patients to enroll in chemotherapy trials to treat their systemic disease. Age, performance status, adjuvant chemotherapy and pre-SRS WBRT were not prognostic factors for improved local control. Noteworthy, pre-SRS WBRT is not a prognostic factor since melanoma and RCC have typically been considered radioresistant. This finding questions the value of the addition of WBRT for treatment of these tumor subtypes. Due to our small event rate, we were unable to differentiate the effect of WBRT for RCC vs. melanoma. Mori *et al*(14) identified age <55 years, lack of active systemic disease and use of chemotherapy and/or immunotherapy after SRS as favorable prognostic factors in multivariate analysis. For melanoma metastases, Mathieu *et al*(28) revealed that predictors of local failure include increased volume of the largest irradiated lesion, increased total irradiation volume, decreased margin, maximum radiation doses and hemorrhagic metastases on univariate analysis and increased total volume of the metastases and hemorrhagic metastases on multivariate analysis. Brown *et al*(30) discovered that adjuvant WBRT improved local control and decreased distant brain failure with 6-month actuarial local control rates of 100 and 85%, respectively, in RCC and melanoma patients. Mori *et al*(14) did not find the addition of WBRT to provide improved local control for melanoma. Our study supports the delay of WBRT for these tumor subtypes since pre-SRS WBRT did not provide any benefit to local control.

### Limitations of the study

There are several limitations as with most retrospective studies and in particular with a challenging patient population required to maintain complete clinical and radiological follow-up. As a result of the small sample size from this single institutional experience, wide confidence intervals were obtained from univariate analysis, making it impossible to perform multivariate analysis.

Since we were mainly interested in local control, where there were multiple metastases in the same individual, each metastasis was analyzed largely independently of the other. We propose that this is a reasonable way to approach the analysis and in fact, this is really no different than what other groups have performed in the past. Although some may argue that multiple metastases in the same individual may respond to SRS similarly based on their similar genetic make-up, clinically we know this is not true. Unfortunately, literature supporting or refuting the multiclonality of multiple brain metastases is not available. Since each metastasis is not a completely independent data point, we accounted for this statistically by stratifying the Cox regression based on the presence of multiple metastases.

In conclusion, SRS is a valuable treatment option for local control of RCC and melanoma brain metastases. Predictors of response, smaller tumor volume and RCC pathology suggest distinct differences in tumor biology and the extent of radioresponse between RCC and melanoma. Concerning local control, pre-SRS WBRT did not provide an additional benefit for either RCC or melanoma subtypes.

## Figures and Tables

**Table I:** Patient demographics.

Description	Value
Age (years)	
Median	52
Range	27–81
Gender	
M	29
F	7
Primary pathology (patients)	
RCC	16
Melanoma	20
Metastasis treated (patients)	
Single	15
Multiple	21
Total number of lesions	103
RCC	41
Melanoma	62
Performance Status	
KPS	
Median	90
Range	70–100
ECOG	
Median	1
Range	0–2
Adjuvant chemotherapy	34
WBRT	
Pre-SRS	56
Follow-up (months)	
Median	6
Range	1–41

RCC, renal cell carcinoma; KPS, Karnofsky performance status; ECOG, Eastern Cooperative Oncology Group; WBRT, whole brain radiation therapy; SRS, stereotactic radiosurgery.

**Table II:** Treatment parameters.

Description	Mean	Median	Range
Prescription dose (Gy)	21.45631068	21	15–25
Conformity index (CI RTOG)	2.5762136	1.93	1.04–9.76
CN	0.49524272	0.51	0.1–0.93
Gradient index	3.241262136	2.96	2.17–7.73
Target volume (cm^3^)	1.43915534	0.4	0.005–13.36
Target min dose (Gy)	20.2515534	20.16	11.62–31.85
Target mean dose (Gy)	32.29067961	32.47	20.44–51.37
No. of shots	4.5145631	2	1–22

RTOG, Radiation Therapy Oncology Group; CI, conformity index.

**Table III:** Actuarial local control (with Kaplan-Meier curve).

6 months	89%
12 months	84%
18 months	76%
24 months	61%

**Table IV:** Univariate cox regression analysis.

Variable	Hazard ratio (95% CI)	P-value
Tumor volume	1.19 (1.05–1.35)	P=0.007
Melanoma vs. RCC	3.48 (1.08–11.23)	P=0.04
Age	1.01 (0.97–1.05)	P=0.7
ECOG score	1.20 (0.46–3.14)	P=0.7
KPS score	0.96 (0.89–1.02)	P=0.19
Chemotherapy	1.17 (0.0.38–3.53)	P=0.8
Pre-SRS WBRT	0.98 (0.30–3.26)	P=0.98

RCC, renal cell carcinoma; ECOG, Eastern Cooperative Oncology Group; KPS, Karnofsky performance status; WBRT, whole brain radiation therapy; SRS, stereotactic radiosurgery.

**Table V:** Published results in the current literature concerning surivival and control in RCC/melanoma cerebral metastases.

Authors	Cases	Local control	Survival	Local recurrence
Brown *et al*(8)	16 RCC; 23 melanoma −83 lesions	100% at 6 months	Median OS, 14.2 months	12%
Buchsbaum *et al*(1)	74 melanoma	NR	Median, 5.5 months	NR
Chang *et al*(10)	44 melanoma, 37 renal, 18 breast, 3 colon, 39 non-small cell lung, 5 sarcoma, 5 other	1 year, 69% response, 2 years, 46% response	NR	NR
Chang *et al*(20)	103 melanoma, 77 RCC, 9 sarcoma − 264 lesions	1 year, 64% RCC; 47% melanoma; 0% sarcoma	Median, 7.5 months; 1 year, 40% RCC; 25% melanoma; 22% sarcoma	NR
Clarke *et al*(9)	27 RCC + melanoma	3 months, 82.8% response 6 months, 77.9% response 9 months, 69.3% response 12 months, 69.3% response 18 months, 55.4% response	NR	26%
Gieger *et al*(11)	12 melanoma, 21 lesions	57%	NR	43%
Goyal *et al*(21)	29 RCC − 66 lesions	NR	Median, 10 months	9%
Halperin and Harisiadis (2)	35 RCC	30%	NR	NR
Kim *et al*(27)	26 lung, 7 kidney, 3 breast, 3 colon − 121 lesions	1 year, 48%	Median, 46 weeks 1 year, 39% response 6 months, 63% response	NR
Lavine *et al*(13)	45 melanoma	97%	Median, 43 months	NR
Lo *et al*(29)	38 melanoma + RCC - 66 lesions	3 months, 87.9% response 6 months, 81.4% response 9 months, 67.9% response 12 months, 67.9% response 18 months, 60.3% response	Corresponding PFS, 55.3, 41.9, 33, 23.3, 13.3%	NR
Maor *et al*(3)	46 RCC	NR	Median, 8	weeks NR
Marko *et al*(23)	19 RCC	95%	Mean, 21.5 months; Median, 13.6 months	NR
Mori *et al*(14)	60 melanoma − 118 lesions	90%	Median, 7 months	11.6%
Mori *et al*(31)	35 RCC − 52 lesions	90%	Median, 11 months	10.2%
Payne *et al*(24)	21 RCC − 37 lesions	100%	NR	0%
Powell *et al*(15)	50 melanoma, 23 RCC, 3 sarcoma	1 year, 77.7% response	Median OS, 5.1 months	NR
Radbill *et al*(16)	51 melanoma − 188 lesions	81%	Median OS, 26 weeks	NR
Schoggl *et al*(25)	23 RCC − 44 lesions	96%	Median, 11 months; 1 year, 48%	NR
Selek *et al*(17)	103 melanoma − 153 lesions	1 year, 49% response	1 year OS, 25.2%	NR
Seung *et al*(18)	55 Melanoma	6 months, 89% response 1 year, 77% response	Median, 35 weeks	NR
Sheehan *et al*(32)	69 RCC − 146 lesions	96%	Median, 15 months	4%
Shuto *et al*(26)	69 RCC	82.6%	Median OS, 9.5 months	NR
Wronski *et al*(4)	119 RCC	NR	6 months, 33.6% response 1 year, 16.8% response 2 years, 5.9% response Median, 3.4 months	NR
Yu *et al*(19)	122 melanoma − 332 lesions	NR	Median, 7 months	NR
Kano *et al*(22)	158 RCC − 531 lesions	92%	OS at 6 months, 60% 12 months, 38% 24 months, 19% Median, 8.2 months	NR

NR, Not reported; RCC, renal cell carcinoma.
